# Supplementary material for: Evaluation of resistance to wheat stem rust and identification of resistance genes in wheat lines from Heilongjiang province
Source: PeerJ. 2021 Feb 9;9:e10580. doi: 10.7717/peerj.10580 (PMC7879953; doi:10.7717/peerj.10580)
Supplement: Supplemental Information 1 [file peerj-09-10580-s001.doc]

| Line | *Sr* Gene | Infection types^a^ | | | *Sr2* | *Sr24* | *Sr25* | *Sr26* | *Sr31* | *Sr38* |
| --- | --- | --- | --- | --- | --- | --- | --- | --- | --- | --- |
|  |  | 21C3CTTTM | 34C0MRGSM | 34C3MTGQM | *Xgwm533* | *Sr24#50* | *Gb* | *Sr26#43* | *SCSS30.2_576_* | *VENTRIUP-LN2* |
|  |  |  | 3 |  |  |  |  |  |  |  |
| ISr5-Ra | *5* | 1 | 4 | 4 | **-** | **-** | **-** | **-** | **-** | **-** |
| CnS_T_mono_der | *21* | 2 | 1 | 2 | **-** | **-** | **-** | **-** | **-** | **-** |
| Vernstine | *9e* | 1- | 1 | 1 | **-** | **-** | **-** | **-** | **-** | **-** |
| ISr7b-Ra | *7b* | 4 | 4 | 4 | **-** | **-** | **-** | **-** | **-** | **-** |
| IS11-Ra | *11* | 3 | 4 | 4 | **-** | **-** | **-** | **-** | **-** | **-** |
| ISr-Ra | *6* | 3 | 4 | 4 | **-** | **-** | **-** | **-** | **-** | **-** |
| ISr8a-Ra | *8a* | 4 | 1+ | 4 | **-** | **-** | **-** | **-** | **-** | **-** |
| CnSr9g | *9g* | 4 | 4 | 4 | **-** | **-** | **-** | **-** | **-** | **-** |
| W2691SrTt-1 | *36* | 3 | ; | 0 | **-** | **-** | **-** | **-** | **-** | **-** |
| W2691Sr9b | *9b* | 4 | 4 | 4 | **-** | **-** | **-** | **-** | **-** | **-** |
| BtS30Wst | *30* | 4 | 1 | 1 | **-** | **-** | **-** | **-** | **-** | **-** |
| Combination Ⅶ | *17+13* | 4 | 0 | ;1- | **-** | **-** | **-** | **-** | **-** | **-** |
| ISr9a-Ra | *9a* | 4 | 4 | 4 | **-** | **-** | **-** | **-** | **-** | **-** |
| ISr9d-Ra | *9d* | 4 | 4 | 4 | **-** | **-** | **-** | **-** | **-** | **-** |
| W2691Sr10 | *10* | 4 | 1 | 1- | **-** | **-** | **-** | **-** | **-** | **-** |
| CnsSrTmp | *Tmp* | 3 | ;1- | ; | **-** | **-** | **-** | **-** | **-** | **-** |
| LcSr24Ag | *24* | 3 | 4 | 4 | **-** | **+** | **-** | **-** | **-** | **-** |
| Sr31/6*LMPG | *31* | ;1 | 1 | ; | **-** | **-** | **-** | **-** | **+** | **-** |
| Federation*4/Kavl | *31* | 1 | 1- | ;1- | **-** | **-** | **-** | **-** | **+** | **-** |
| Trident | *38* | ; | 1 | 1 | **-** | **-** | **-** | **-** | **-** | **+** |
| Hope | *2* | 3 |  | 3 | **+** | **-** | **-** | **-** | **-** | **-** |
| SwSr22T.B. | *22* | 1+ | 2 | 3+ | **-** | **-** | **-** | **-** | **-** | **-** |
| Agatha/9*LMPG | *25* | 3 | 3 | 2 | **-** | **-** | **+** | **-** | **-** | **-** |
| Eagle | *26* | 0 | 0 | 0 | **-** | **-** | **-** | **+** | **-** | **-** |
| 73,214,3-1/9*LMH? | *27* | 1 | 3+ | 2 | **-** | **-** | **-** | **-** | **-** | **-** |
| ER 5155 | *32* | 3 | 2 | 4 | **-** | **-** | **-** | **-** | **-** | **-** |
| Tetra Canthatch/A? | *33* | 0 | 1 | 0 | **-** | **-** | **-** | **-** | **-** | **-** |
| Mq(2)5XG2919 | *35* | 1 | 0 | 1 | **-** | **-** | **-** | **-** | **-** | **-** |
| W3563 | *37* | 1 | 2 | 1 | **-** | **-** | **-** | **-** | **-** | **-** |
| L6082 | *39* | 1+ | 3 | 4 | **-** | **-** | **-** | **-** | **-** | **-** |
| DAS15 | *47* | ; | ; | 0 | **-** | **-** | **-** | **-** | **-** | **-** |
| Leeds | *9e*, *13*, *+* | 1 | 1- | ; | **-** | **-** | **-** | **-** | **-** | **-** |
| Siouxland | *24+31* | 0; | ;1- | 0; | **-** | **+** | **-** | **-** | **+** | **-** |
| Sisson | *31+36* | ; | 0 | 0 | **-** | **-** | **-** | **-** | **+** | **-** |
| Fed/SrTt3 | *Tt3* | 0 | 0 | 0 |  |  |  |  |  |  |
| McNair 701 | *McN* | 4 | 4 | 4 | **-** | **-** | **-** | **-** | **-** | **-** |
| Little Club | *-* | 4 | 4 | 4 | **-** | **-** | **-** | **-** | **-** | **-** |

^a^ IT: infection types scored in the greenhouse seedling tests were based on a 0-to-4 scale (Stakman et al. 1962) where ITs ;, 0, 1, or 2 were considered resistant and ITs 3 or 4 susceptible; and symbols + and – indicated slightly larger and smaller pustule sizes, respectively.
